# Effort-reward imbalance and the mental health of middle managers in Europe

**DOI:** 10.1093/eurpub/ckac131.275

**Published:** 2022-10-25

**Authors:** M Vos, D De Moortel, C Vanroelen

**Affiliations:** SOCI, Vrije Universiteit Brussel, Elsene, Belgium

## Abstract

**Background:**

According to the Neo-Marxist Class Theory, supervisors’ health is at risk due to their position of authority without strategic power. We investigate how the interaction between the class location and an Effort-Reward Imbalance (ERI) is related to mental health risk, including gender differences and mediation by work-life balance.

**Methods:**

From the 6th wave of the European Working Conditions Survey, we selected workers aged 15 to 64 of the 28 European Member States (pre-Brexit). ERI was measured with 18 proxies for the ERI Questionnaire items. For mental health, the WHO-5 well-being index was used. Relationships were analyzed using linear regression models.

**Results:**

We found evidence for the relationship between ERI and mental health of European employees (ß = -0.641, p < .001), partially mediated by work-life balance. Contrary to previous NMSC studies, we did not find worse mental health for supervisors. The vulnerability for ERI increases with class position (supervisors ß = -0.703; topmanagers ß = -1.099), with supervisors showing a higher mean ERI (subordinates M = 0.445; supervisors M = 0.459; topmanagers M = 0.437, p < .001). The mental health of female supervisors appears more vulnerable to ERI than men’s.

**Conclusions:**

Our findings show that mental health risks of supervisors become apparent especially in situations where esteem, job security and promotion opportunities do not match the status expectations of the position. A labor market policy that encourages organizations to have those tasks performed by their own permanent employees (as opposed to outsourcing them), with a focus on a healthy work-life balance and fair remuneration, can benefit the mental health of this group of employees.

**Key messages:**

• The mental health of employees in higher positions of authority is more vulnerable to situations of effort-reward imbalance.

• European labor market policies focused on security for employees, rather than flexibility for employers, can reduce mental illness among the European middle managers and subordinates.

